# Comparative Evaluation of Fixed Versus Removable Tongue-Thrust Habit-Breaking Appliances in the Management of Anterior Open Bite in Pediatric Patients

**DOI:** 10.7759/cureus.111708

**Published:** 2026-06-29

**Authors:** Srikanth Reddy Karra, Sukanta Kumar Dey, Parul Bhatnagar, Meena Sharan, Shalini Krishna, Khoda Opi

**Affiliations:** 1 Department of Oral Health Sciences, Temple University Kornberg School of Dentistry, Philadelphia, USA; 2 Department of Orthodontics, Awadh Dental College and Hospital, Jamshedpur, IND; 3 Department of Pedodontics and Preventive Dentistry, Teerthanker Mahaveer Dental College and Research Centre, Teerthanker Mahaveer University, Moradabad, IND; 4 Department of Pedodontics and Preventive Dentistry, Seema Dental College and Hospital, Rishikesh, IND; 5 Department of Pedodontics and Preventive Dentistry, The Oxford Dental College, Bengaluru, IND

**Keywords:** anterior open bite, orthodontic appliances, pediatric dentistry, tongue habit, tongue thrust

## Abstract

Introduction: Anterior open bite associated with tongue-thrusting habit is a common malocclusion in children that may adversely affect dentofacial development, oral function, and esthetics. Habit-breaking appliances are frequently used to eliminate tongue-thrusting behavior and facilitate the correction of anterior open bites. However, limited evidence exists regarding the comparative effectiveness of fixed and removable tongue-thrust habit-breaking appliances. This study aimed to compare the effectiveness of fixed and removable tongue-thrust habit-breaking appliances in managing anterior open bites in pediatric patients.

Materials and methods: This retrospective observational comparative study included 100 pediatric patients aged 7-14 years diagnosed with an anterior open bite associated with a tongue-thrusting habit. The patients were divided into two groups: fixed (n = 50) and removable (n = 50) appliance groups. Demographic and clinical data were extracted from patient records. The outcome measures included reduction in anterior open bite, percentage correction, treatment duration, complete open-bite closure, and predictors of successful treatment. Statistical analyses were performed using appropriate parametric and regression tests with a significance level of p < 0.05.

Results: The two groups were comparable at baseline, with no significant differences in age, sex distribution, baseline open bite severity, duration of habit, or follow-up period (p > 0.05). Both treatment modalities produced significant reductions in the anterior open bite (p < 0.001). The fixed appliance group demonstrated a greater mean reduction in open bite (2.9 ± 1.0 mm) than the removable appliance group (1.9 ± 0.8 mm) (p < 0.001). The percentage correction was significantly higher in the fixed appliance group (76.3 ± 12.4%) than in the removable appliance group (51.4 ± 15.7%) (p < 0.001). Treatment duration was significantly shorter with fixed appliances (10.2 ± 2.1 months) than with removable appliances (11.8 ± 2.6 months) (p = 0.001). Complete open-bite closure was achieved in 35 (70.0%) and 20 (40.0%) patients in the fixed and removable appliance groups, respectively (p = 0.003). Fixed appliance therapy was the strongest independent predictor of successful open bite closure (odds ratio (OR) = 4.00; 95% confidence interval (CI): 1.75-9.14; p = 0.001).

Conclusion: Both fixed and removable tongue-thrust habit-breaking appliances were effective in reducing anterior open bites in pediatric patients. However, fixed appliances achieved significantly greater correction, higher rates of complete closure, and shorter treatment durations. Fixed appliance therapy demonstrated superior clinical effectiveness and was the strongest predictor of successful treatment outcomes. These findings support the use of fixed habit-breaking appliances as a preferred treatment option for growing children with tongue-thrust-associated anterior open bites.

## Introduction

Anterior open bite is a common malocclusion in the pediatric population, characterized by the absence of vertical overlap between the maxillary and mandibular anterior teeth when the posterior teeth are in occlusion [1]. The etiology of anterior open bite is multifactorial and may involve genetic, skeletal, dental, and functional factors. Among the various contributing factors, tongue-thrusting is recognized as one of the most significant functional causes of anterior open bite in growing children [2,3]. Persistent forward tongue placement during swallowing, speech, and rest can exert abnormal forces on the dentition, resulting in impaired eruption patterns, proclination of anterior teeth, and maintenance or worsening of open bite malocclusion [4].

Early interception of tongue-thrusting habits is essential to prevent the progression of malocclusion and promote normal dentofacial development. Habit-breaking appliances are commonly employed as interceptive orthodontic measures to eliminate aberrant tongue postures and facilitate the spontaneous correction of anterior open bites [5,6]. These appliances are broadly classified into fixed and removable types. Fixed tongue cribs offer continuous action independent of patient cooperation, whereas removable appliances provide ease of maintenance and improved oral hygiene but require consistent patient compliance for successful outcomes [7].

Several studies have reported favorable outcomes with both fixed and removable tongue-thrust appliances; however, evidence comparing their relative effectiveness remains limited and occasionally contradictory [7]. Differences in treatment duration, patient compliance, habit cessation rates, and the extent of open bite correction may influence appliance selection in clinical practice. Therefore, a direct comparison of these treatment modalities is necessary to guide evidence-based decision-making and optimize treatment outcomes in pediatric patients. This study aimed to retrospectively compare the effectiveness of fixed and removable tongue-thrust habit-breaking appliances in managing anterior open bites in pediatric patients. The objectives were to evaluate and compare the reduction in anterior open bite, percentage correction achieved, treatment duration required for correction, and rate of complete habit cessation and open-bite closure between the two appliance types, as well as to identify factors associated with successful treatment outcomes.

## Materials and methods

Study design

This retrospective observational comparative study was conducted in the Department of Pedodontics and Preventive Dentistry, Kothiwal Dental College and Research Centre, Moradabad, India, on the archival records of patients from January 2012 to January 2021, following approval from the Institutional Ethics Committee (IEC). As the study involved a retrospective analysis of existing patient records with no direct patient contact or intervention, the IEC granted a waiver of informed consent. All patient identifiers were removed before data extraction, and confidentiality and anonymity were strictly maintained throughout data collection, analysis, and reporting, in accordance with institutional guidelines and the principles of the Declaration of Helsinki.

This study involved a systematic review and analysis of patient records maintained in departmental archives. Clinical records from the preceding years were screened to identify eligible patients who had undergone treatment for an anterior open bite associated with the tongue-thrusting habit using either fixed or removable habit-breaking appliances. Patient records, clinical images, treatment charts, and follow-up documentation maintained by the departments were used for data collection. All data extraction and analysis were performed within the department, following strict confidentiality protocols.

Study population

The study population comprised pediatric patients (age 7-10 years) diagnosed with an anterior open bite associated with a tongue-thrusting habit who received interceptive orthodontic treatment using tongue-thrust habit-breaking appliances. Only patients with complete pre-treatment and post-treatment records and adequate follow-up documentation were considered eligible for inclusion in the study.

Sample size estimation

Sample size estimation was performed using the G*Power software version 3.1.9.7 (Heinrich Heine University Düsseldorf, Düsseldorf, Germany). For comparison between two independent treatment groups, an independent samples t-test was selected with a two-tailed significance level of 5% and statistical power of 80%. Based on a previously reported effect size (Cohen’s d = 0.60) for open-bite reduction, the minimum required sample size was calculated as 45 participants per group [8]. To compensate for incomplete records and potentially missing data, the sample size was increased to 50 patients in each group, resulting in a total sample size of 100 participants.

Study groups

**Figure 1:**
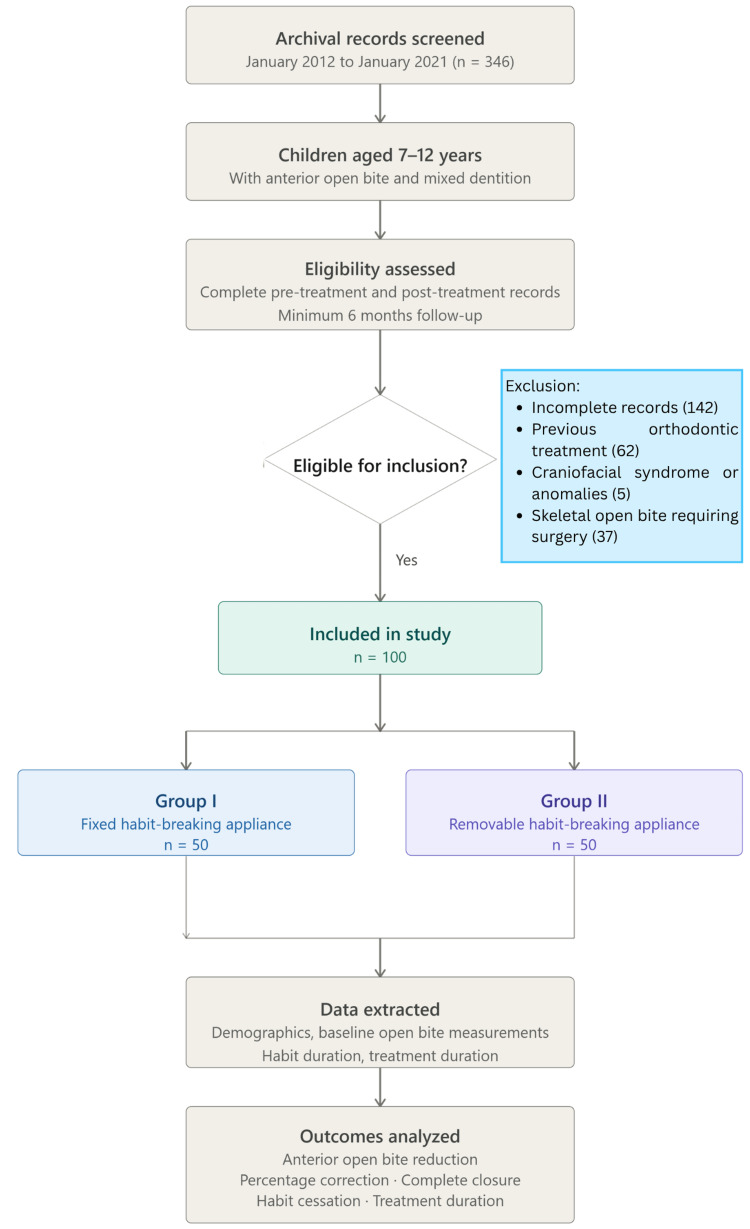
Study flow diagram. Figure made by using Canva (Version 2.0, Canva Pty Ltd., Sydney, NSW, Australia). No artificial intelligence (AI) tool was used to create the image.

Eligible participants were categorized into two treatment groups according to the appliances used for habit interception. Group I (n = 50) comprised patients treated with fixed tongue-thrust habit-breaking appliances, such as fixed tongue cribs. Group II (n = 50) comprised patients treated with removable tongue-thrust habit-breaking appliances (Figure 1).

Eligibility criteria

**Figure 2:**
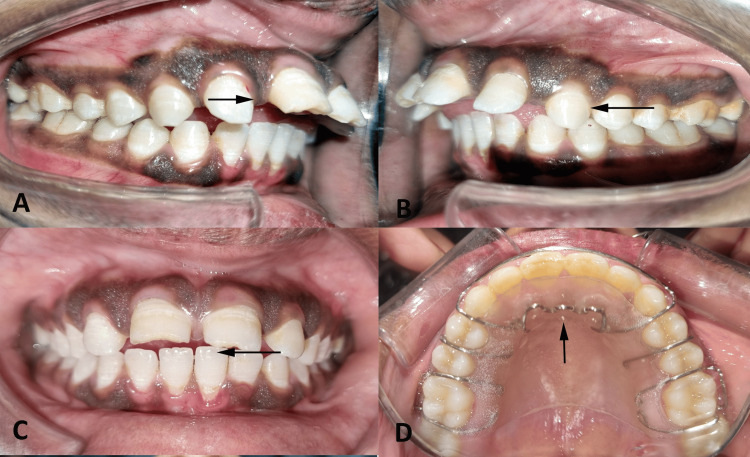
Dentoalveolar anterior open bite (A) as viewed from right lateral view, (B) as viewed from left lateral view, (C) as viewed from frontal view, (D) treated with removable tongue crib. Original images of the patient from the study.

Children aged 7-10 years in the early mixed dentition period presenting with dentoalveolar anterior open bite associated with tongue-thrusting habit were included in the study. The tongue-thrusting pattern was considered an adaptive habit secondary to prolonged non-nutritive sucking habits and was clinically documented in the patient records by the treating orthodontist. Only patients exhibiting a dental anterior open bite with a normal skeletal relationship (skeletal Class I pattern) and treated exclusively with either fixed or removable tongue-thrust habit-breaking appliances were eligible for inclusion (Figure 2). The appliances were given as a part of interceptive orthodontics, where removal of habit in early mixed dentition could lead to spontaneous closure of the anterior open bite by eruption of incisors.

Availability of complete pre-treatment and post-treatment clinical records, including documented measurements of anterior open bite and a minimum follow-up period of six months after appliance placement, was mandatory. Patients with craniofacial syndromes, congenital craniofacial anomalies, skeletal discrepancies, skeletal anterior open bite requiring comprehensive orthodontic treatment, or any other dentofacial deformity affecting vertical occlusion were excluded. Patients with a history of previous orthodontic treatment, incomplete records, enlarged adenoids, missing follow-up data, or persistence of other oral habits, such as thumb sucking, digit sucking, lip habits, and mouth breathing during treatment, were also excluded.

Record screening and patient selection

All available patient records during the designated study period were screened by one trained orthodontist (SKD) and one trained pedodontist (SK). Records were initially reviewed for diagnosis, treatment modalities, and completeness of documentation. Patients who fulfilled all the inclusion criteria and none of the exclusion criteria were enrolled in the study. A unique study identification number was assigned to each participant to ensure anonymity and confidentiality of the data.

Calibration of investigators

Before data collection, the two investigators responsible for record evaluation underwent a calibration exercise to standardize the assessment. Twenty patient records that were not included in the final study sample were randomly selected for training purposes. The investigators independently assessed the anterior open bite measurements, appliance type, treatment duration, and treatment outcomes. Any discrepancies were discussed with a pedodontist (PB) until consensus was reached. Standardized operational definitions and measurement protocols were established before the main study commenced.

Reliability assessment

To assess intra-examiner reliability, 20 randomly selected patient records were re-evaluated by the primary investigator (SKD) two weeks after the initial assessment without access to previous measurements. Inter-examiner reliability was assessed by comparing the measurements independently recorded by the two calibrated investigators (SKD and SK). Reliability analysis was performed under the supervision of a pedodontist (PB). For continuous variables, including anterior open bite measurements, the intraclass correlation coefficient (ICC) was calculated, and demonstrated excellent agreement (ICC > 0.85). For categorical variables, including appliance type, complete habit cessation, and open bite closure status, Cohen's kappa coefficient showed excellent agreement (κ > 0.85). These findings confirmed high intra- and inter-examiner reliability and ensured the consistency and reproducibility of the collected data.

Data collection procedure

Data were extracted from the patient records using a standardized data collection form specifically developed for this study. Clinical charts, orthodontic records, treatment notes, and follow-up documentation were systematically reviewed. All collected data were entered into a password-protected electronic database and verified for accuracy by an independent investigator. Demographic information included age at the initiation of treatment and sex. The clinical variables included baseline anterior open bite severity, duration of tongue-thrust habit, type of habit-breaking appliance used, treatment duration, and follow-up duration.

Outcome variables

The primary outcome variable was the reduction in anterior open bite, measured in millimeters, from baseline to post-treatment. The secondary outcome variables included the percentage correction of anterior open bite, complete closure of anterior open bite, complete cessation of tongue-thrust habit, and duration of treatment required to achieve correction.

Measurement of anterior open bite

Anterior open bite was measured from clinical records as the vertical distance between the incisal edge of the most erupted maxillary central incisor and the corresponding mandibular central incisor when the posterior teeth were in maximum intercuspation. The measurements were recorded in millimeters. Baseline measurements obtained before appliance placement and post-treatment measurements recorded at the completion of treatment were used for the analysis. The reduction in anterior open bite was calculated by subtracting the post-treatment measurements from baseline measurements. Percentage correction was determined using the following formula: 

\[ \text{Percentage correction (%)} = \frac{\text{Baseline open bite} - \text{Post-treatment open bite}} {\text{Baseline open bite}} \times 100 \]

Assessment of habit cessation

Habit cessation was assessed based on clinical records documenting the elimination of tongue thrusting during swallowing and resting tongue posture at the end of treatment. Patients were categorized as either exhibiting complete habit cessation or persistent habit according to the clinical assessment of the treating orthodontist.

Data management and confidentiality

All patient identifiers, including names, registration numbers, and contact details, were removed before data extraction. Each participant was assigned a unique code. Electronic data were stored on password-protected computers accessible only to the research team members. Hard-copy records were secured within departmental archives throughout the study period.

Statistical analysis

Statistical analyses were performed by a blinded statistician (SRK) using THE IBM SPSS Statistics for Windows, Version 25.0 (IBM Corp., Armonk, New York, USA). The data were initially screened for completeness and accuracy. Continuous variables were tested for normality using the Shapiro-Wilk test. Normally distributed continuous variables are expressed as mean ± standard deviation, whereas categorical variables are presented as frequencies and percentages. Baseline demographic and clinical characteristics between groups were compared using independent samples t-tests for continuous variables and chi-square tests for categorical variables. Within-group changes in anterior open bite from baseline to post-treatment were analyzed using paired t-tests. Between-group comparisons of open bite reduction, percentage correction, and treatment duration were performed using independent samples t-tests.

Factors associated with successful open-bite closure were investigated using univariable logistic regression analysis. Variables demonstrating a significance level of p < 0.10 in the univariable analysis were subsequently entered into a multivariable logistic regression model to identify independent predictors of successful treatment outcomes. The results are expressed as odds ratios (ORs) with 95% confidence intervals (CIs). A p-value of < 0.05 was considered statistically significant for all analyses.

## Results

A total of 100 pediatric patients diagnosed with an anterior open bite associated with a tongue-thrusting habit were included in this study. The participants were equally distributed into the fixed (n = 50) and removable (n = 50) appliance groups. The demographic characteristics of the study participants are shown in Table 1. No statistically significant differences were observed between the two groups with respect to age, sex distribution, or baseline anterior open bite severity (p > 0.05). The mean age was 9.4 ± 2.1 years in the fixed appliance group and 9.6 ± 2.3 years in the removable appliance group. These findings indicate that the groups were comparable at the baseline.

**Table 1:** Demographic characteristics of the study participants. Data presented as mean ± standard deviation (SD) or n (%), independent samples t-test used for continuous variables, chi-square (χ²) test used for categorical variables, p < 0.05 considered statistically significant.

Variable	Fixed appliance (n = 50)	Removable appliance (n = 50)	Test statistic	p-value
Age (years), mean ± SD	9.4 ± 2.1	9.6 ± 2.3	t = -0.456	0.649
Sex (male), n (%)	26 (52)	24 (48)	χ² = 0.160	0.689
Sex (female), n (%)	24 (48)	26 (52)		
Baseline open bite (mm), mean ± SD	3.8 ± 1.2	3.7 ± 1.1	t = 0.433	0.666

The baseline clinical characteristics are presented in Table 2. The mean duration of the tongue-thrusting habit was 4.2 ± 1.5 years in the fixed appliance group and 4.4 ± 1.6 years in the removable appliance group. Similarly, the follow-up duration was comparable between the groups. No significant differences were observed in the baseline clinical parameters (p > 0.05). 

**Table 2:** Comparison of baseline clinical characteristics between the study groups. Data presented as mean ± standard deviation (SD), independent samples t-test used for group comparisons, p < 0.05 considered statistically significant.

Variable	Fixed appliance (n = 50)	Removable appliance (n = 50)	Test statistic	p-value
Open bite severity (mm), mean ± SD	3.8 ± 1.2	3.7 ± 1.1	t = 0.433	0.666
Duration of habit (years), mean ± SD	4.2 ± 1.5	4.4 ± 1.6	t = -0.641	0.523
Follow-up duration (months), mean ± SD	12.4 ± 2.3	12.1 ± 2.5	t = 0.624	0.534

The within-group comparison of anterior open-bite reduction is presented in Table 3. Both treatment modalities demonstrated significant improvements following therapy. In the fixed appliance group, the mean anterior open bite decreased from 3.8 ± 1.2 mm to 0.9 ± 0.7 mm, resulting in a mean reduction of 2.9 mm (p < 0.001). In the removable appliance group, the mean open bite decreased from 3.7 ± 1.1 mm to 1.8 ± 0.9 mm, corresponding to a mean reduction of 1.9 mm (p < 0.001).

**Table 3:** Within-group comparison of anterior open bite reduction following treatment. Data presented as mean ± standard deviation (SD), paired t-test used for within-group comparisons between baseline and post-treatment measurements, *p < 0.05 considered statistically significant.

Group	Baseline open bite (mm) mean ± SD	Post-treatment open bite (mm) mean ± SD	Mean reduction (mm)	Paired t-value	p-value
Fixed appliance	3.8 ± 1.2	0.9 ± 0.7	2.9	t = 14.82	< 0.001*
Removable appliance	3.7 ± 1.1	1.8 ± 0.9	1.9	t = 9.86	< 0.001*

A comparison of the treatment outcomes between the two groups is summarized in Table 4. The fixed appliance group exhibited a significantly greater reduction in anterior open bite (2.9 ± 1.0 mm) than the removable appliance group (1.9 ± 0.8 mm) (p < 0.001). The percentage correction was also significantly higher in the fixed appliance group (76.3 ± 12.4%) than in the removable appliance group (51.4 ± 15.7%) (p < 0.001). Furthermore, treatment duration was significantly shorter in patients treated with fixed appliances (10.2 ± 2.1 months) than in those treated with removable appliances (11.8 ± 2.6 months) (p = 0.001).

**Table 4:** Comparison of treatment outcomes between fixed and removable tongue-thrust habit-breaking appliances. Data presented as mean ± standard deviation (SD), independent samples t-test used for between-group comparisons, *p < 0.05 considered statistically significant.

Outcome variable	Fixed appliance (n = 50)	Removable appliance (n = 50)	Test statistic	p-value
Reduction in open bite (mm), mean ± SD	2.9 ± 1.0	1.9 ± 0.8	t = 5.477	< 0.001*
Percentage correction (%), mean ± SD	76.3 ± 12.4	51.4 ± 15.7	t = 8.783	< 0.001*
Treatment duration (months), mean ± SD	10.2 ± 2.1	11.8 ± 2.6	t = -3.376	0.001*

The rates of complete anterior open-bite closure are presented in Table 5. Complete closure was achieved in 35 (70%) and 20 (40%) patients in the fixed and removable appliance groups, respectively. The difference between the groups was statistically significant (p = 0.003), indicating superior treatment success with fixed appliances.

**Table 5:** Comparison of complete anterior open-bite closure between the study groups. Data presented as n (%), chi-square (χ²) test used for comparison of categorical variables, *p < 0.05 considered statistically significant, χ² = chi-square statistic.

Closure status	Fixed appliance (n = 50) n (%)	Removable appliance (n = 50) n (%)	χ² value	p-value
Complete closure	35 (70%)	20 (40%)	9.09	0.003*
Incomplete closure	15 (30%)	30 (60%)		

The predictors of successful open-bite closure identified through multivariable logistic regression analysis are shown in Table 6. Appliance type emerged as the strongest independent predictor of successful treatment outcomes. Patients treated with fixed appliances demonstrated four times greater odds of achieving successful closure than those treated with removable appliances (OR = 4.00; p = 0.001). A longer treatment duration was also significantly associated with successful closure (OR = 1.25; p = 0.024). Age showed borderline significance, whereas sex and baseline open bite severity were not significant predictors.

**Table 6:** Multivariable logistic regression analysis of predictors of successful anterior open bite closure. Data are presented as regression coefficient (β), standard error, odds ratio, and 95% confidence interval (CI). Multivariable logistic regression analysis was performed to identify independent predictors of successful open-bite closure. *p < 0.05 is considered statistically significant.

Variable	β coefficient	Standard error	Odds ratio (95% CI)	p-value
Appliance type (fixed vs. removable)	1.386	0.421	4.00 (1.75-9.14)	0.001*
Age (per year increase)	-0.182	0.095	0.83 (0.69–-1.00)	0.055
Sex (male vs. female)	0.105	0.389	1.11 (0.52-2.38)	0.787
Baseline open bite severity (per mm)	-0.095	0.164	0.91 (0.66-1.25)	0.562
Treatment duration (per month)	0.223	0.099	1.25 (1.03-1.52)	0.024*

## Discussion

Anterior open bite associated with tongue-thrusting habits is a common dentofacial problem during childhood and can adversely affect esthetics, speech, swallowing patterns, and overall occlusal development. Interceptive orthodontic treatment aimed at eliminating the etiological habit and promoting spontaneous correction of malocclusion is therefore essential. Habit-breaking appliances, particularly tongue cribs, have been widely employed for this purpose; however, evidence comparing the effectiveness of fixed and removable appliances is limited [7]. The present retrospective comparative study evaluated the treatment outcomes associated with fixed and removable tongue-thrust habit-breaking appliances in pediatric patients with anterior open bite.

The baseline demographic and clinical characteristics were comparable between the two treatment groups, indicating adequate matching and minimization of potential confounding influences. No significant differences were observed in age, sex distribution, baseline open bite severity, duration of the habit, or follow-up period. These findings strengthen the validity of subsequent comparisons and suggest that the differences observed in treatment outcomes were primarily attributable to the type of appliance used rather than baseline disparities.

Both fixed and removable appliances produced significant reductions in anterior open bite after treatment. The fixed appliance group demonstrated a mean reduction of 2.9 mm, whereas the removable appliance group showed a mean reduction of 1.9 mm. These findings confirm that elimination of the tongue-thrusting habit plays a critical role in facilitating the correction of anterior open bites [4]. The observed improvements are consistent with previous investigations that reported favorable dentoalveolar changes following tongue crib therapy [9-11]. By preventing anterior tongue positioning during swallowing and at rest, habit-breaking appliances reduce the abnormal muscular forces acting on the anterior teeth, thereby permitting vertical eruption of the incisors and spontaneous closure of the open bite [12].

A major finding of the present study was the significantly greater treatment effectiveness associated with fixed appliances than with removable appliances. Fixed appliances achieved significantly greater open-bite reduction, higher percentage correction, and shorter treatment duration. These findings are in agreement with those of previous studies that reported superior dentoskeletal improvements in mixed dentition patients treated with fixed tongue cribs [8,13]. The authors suggested that continuous appliance action and independence from patient cooperation substantially contributed to treatment success. Similar observations have been reported by Barnawi et al. [7], who demonstrated significant dentoalveolar improvements and enhanced vertical control following fixed crib therapy in growing patients.

The superior performance of fixed appliances observed in the present study can be explained by their continuous therapeutic effects. Unlike removable appliances, fixed cribs remain in place throughout the treatment period and function regardless of patient motivation or compliance. Pediatric patients frequently exhibit inconsistent appliance wear, particularly during school hours or social situations, which may reduce the effectiveness of the removable appliances. Consequently, fixed appliances provide a more reliable interruption of abnormal tongue posture and swallowing patterns, resulting in a greater correction of the associated malocclusion [14].

The findings of the present study differ from those reported by Meng et al. [15] in their systematic review and meta-analysis, which concluded that bonded lingual spurs, fixed palatal cribs or spurs, and removable palatal cribs demonstrated comparable effectiveness in the early treatment of anterior open bites. The authors observed no significant differences in overbite correction between the fixed and removable appliances. Slaviero et al. [16] also reported that both fixed and removable palatal cribs were similarly effective in correcting anterior open bite, producing comparable increases in overbite after one year of treatment. In contrast, the present study demonstrated significantly greater open-bite reduction and higher complete closure rates with fixed appliances, suggesting a potential advantage of fixed therapy in clinical practice. In contrast, the present study demonstrated significantly greater open-bite reduction, a higher percentage correction, shorter treatment duration, and a higher rate of complete closure with fixed appliances. These differences may be attributed to variations in the study design, sample characteristics, treatment protocols, follow-up duration, and outcome measures. Furthermore, the meta-analysis included only four studies and reported low quality of evidence, suggesting that additional well-designed clinical studies are required to establish definitive recommendations regarding the comparative effectiveness of different habit-breaking appliances [15].

Another important finding was the significantly higher rate of complete open-bite closure achieved with fixed appliances. These results further support the superiority of fixed appliances in achieving clinically meaningful treatment results. Previous studies have similarly reported higher success rates and more stable corrections when patient compliance is not a limiting factor [7,17]. The ability of fixed appliances to maintain continuous control over tongue posture likely contributes to a more favorable environment for the eruption and alignment of the anterior dentition [17].

Multivariable logistic regression analysis identified the appliance type as the strongest independent predictor of successful open-bite closure. Patients treated with fixed appliances demonstrated fourfold higher odds of successful correction than those treated with removable appliances [7]. This finding highlights the importance of appliance selection in interceptive orthodontic treatment planning. Treatment duration was also a significant predictor of successful closure, indicating that adequate treatment continuation is necessary to achieve stable habit elimination and dentoalveolar adaptation [18]. Although younger age showed borderline significance, sex and baseline open bite severity did not significantly influence treatment success, suggesting that appliance effectiveness may be more important than demographic factors in determining the treatment outcomes.

An important consideration in interpreting the present findings is the etiological relationship between tongue thrusting and anterior open bite. The study specifically included pediatric patients diagnosed with anterior open bite associated with tongue-thrusting habit and excluded patients with skeletal open bite requiring comprehensive orthodontic treatment. Therefore, the open bites evaluated were predominantly dentoalveolar in nature rather than skeletal. Although the relationship between tongue thrust and anterior open bite remains complex and potentially bidirectional, clinical evidence suggests that persistent tongue-thrusting behavior can contribute to the development and maintenance of dental open bite by exerting abnormal forces on the anterior dentition and preventing normal vertical eruption of incisors [3,6-8]. Conversely, an existing open bite may also facilitate adaptive forward tongue posture, thereby perpetuating the habit. In the present study, treatment was directed toward the elimination of the tongue-thrusting habit through habit-breaking appliances [8]. The significant reduction and closure of the open bite observed following habit interception suggest that removal of the abnormal functional influence allowed spontaneous dentoalveolar adaptation and vertical eruption of the anterior teeth [3]. Consequently, the correction achieved in the present cohort is most likely attributable to elimination of the tongue-thrust habit and the resulting normalization of tongue posture rather than skeletal modification, which was not the objective of the treatment and was outside the scope of the study population.

The findings of the present study have important clinical implications. Fixed tongue-thrust habit-breaking appliances may be considered the preferred treatment option for pediatric patients with anterior open bite, particularly when concerns regarding patient compliance exist. The greater magnitude of correction, shorter treatment duration, and higher likelihood of complete closure observed with fixed appliances may facilitate more predictable treatment outcomes and reduce the need for subsequent comprehensive orthodontic interventions. Early interception of tongue-thrusting habits through fixed appliances may contribute to improved occlusal development and long-term stability.

Despite these encouraging findings, several limitations of this study should be acknowledged. The retrospective study design limits the ability to establish definitive causal relationships and is inherently subject to selection bias. Data were obtained from a single institution, which may restrict the generalizability of the results to broader populations. Patient compliance could not be objectively quantified, particularly in the removable appliance group, and this factor may have influenced the treatment outcomes. In addition, long-term post-treatment stability and relapse rates were not evaluated. Future prospective randomized controlled trials with larger multicenter samples and extended follow-up periods are recommended to validate the present findings and assess the long-term effectiveness and stability of fixed and removable tongue-thrust habit-breaking appliances.

## Conclusions

Both fixed and removable tongue-thrust habit-breaking appliances were effective in reducing anterior open bite in pediatric patients with tongue-thrusting habits. However, fixed appliances demonstrated significantly greater open-bite reduction, a higher percentage correction, shorter treatment duration, and a higher rate of complete open-bite closure compared with removable appliances. Appliance type emerged as the strongest predictor of successful treatment outcome. These findings suggest that fixed tongue-thrust habit-breaking appliances provide a more predictable and efficient approach for the management of anterior open bite in growing children, particularly when patient compliance may be a concern.
